# Drug Authorization for Sofosbuvir/Ledipasvir (Harvoni) for Chronic HCV Infection in a Real-World Cohort: A New Barrier in the HCV Care Cascade

**DOI:** 10.1371/journal.pone.0135645

**Published:** 2015-08-27

**Authors:** Albert Do, Yash Mittal, AnnMarie Liapakis, Elizabeth Cohen, Hong Chau, Claudia Bertuccio, Dana Sapir, Jessica Wright, Carol Eggers, Kristine Drozd, Maria Ciarleglio, Yanhong Deng, Joseph K. Lim

**Affiliations:** 1 Department of Internal Medicine, Yale University School of Medicine, New Haven, CT, United States of America; 2 Yale Liver Center, Section of Digestive Diseases, New Haven, CT, United States of America; 3 Liver Transplantation Program, Yale-New Haven Hospital, New Haven, CT, United States of America; 4 Apothecary and Wellness Center, Yale-New Haven Hospital, New Haven, CT, United States of America; University of North Carolina School of Medicine, UNITED STATES

## Abstract

**Background:**

New treatments for hepatitis C (HCV) infection hold great promise for cure, but numerous challenges to diagnosing, establishing care, and receiving therapy exist. There are limited data on insurance authorization for these medications.

**Materials and Methods:**

We performed a retrospective chart review of patients receiving sofosbuvir/ledipasvir (SOF/LED) from October 11-December 31, 2014 to determine rates and timing of drug authorization. We also determined predictors of approval, and those factors associated with faster decision and approval times.

**Results:**

Of 174 patients prescribed HCV therapy during this period, 129 requests were made for SOF/LED, of whom 100 (77.5%) received initial approval, and an additional 17 patients (13.9%) ultimately received approval through the appeals process. Faster approval times were seen in patients with Child-Pugh Class B disease (14.4 vs. 24.7 days, p = 0.048). A higher proportion of patients were initially approved in those with Medicare/Medicaid coverage (92.2% vs. 71.4%, p = 0.002) and those with baseline viral load ≥6 million IU/mL (84.1% vs. 62.5%, p = 0.040). Linear regression modeling identified advanced fibrosis, high Model of End Stage Liver Disease (MELD) score, and female gender as significant predictors of shorter decision and approval times. On logistic regression, Medicare/Medicaid coverage (OR 5.96, 95% CI 1.66–21.48) and high viral load (OR 4.52, 95% CI 1.08–19.08) were significant predictors for initial approval.

**Conclusions:**

Early analysis of real-world drug authorization outcomes between October-December 2014 reveals that nearly one in four patients are initially denied access to SOF/LED upon initial prescription, although most patients are eventually approved through appeal, which delays treatment initiation. Having Medicare/Medicaid and advanced liver disease resulted in a higher likelihood of approval as well as earlier decision and approval times. More studies are needed to determine factors resulting in higher likelihood of denial and to evaluate approval rates and times after implementation of restrictive prior authorization guidelines.

## Introduction

Treatment of chronic hepatitis C (HCV) infection in the United States has been revolutionized with the development of novel direct-acting antiviral (DAA) therapies. DAA therapy has demonstrated better tolerability, adherence, as well as rates of sustained virologic response (SVR) and cure compared to antecedent interferon (IFN)-based therapies [1–4]. This advance has expanded the population of individuals with HCV infection who are potentially treatable. Owing to its efficacy, the American Association for the Study of Liver Disease (AASLD) and the Infectious Disease Society of America (IDSA) have modified their recommendations to include the combination of sofosbuvir and ledipasvir (SOF/LED) as first-line therapy for HCV genotype-1 infection, the most prevalent strain seen in the United States [3, 5–8].

However, care provision requires successful completion of numerous steps along a care continuum [9]. It has been recently estimated that only 16% of chronic HCV-infected individuals are prescribed antiviral treatment and only 9% achieve SVR [10] although this represents data from the interferon era and precedes the advent of all-oral anti-HCV regimens. The concept of a care cascade (diagnosis, linkage to care, retention in care, prescription of antiretroviral therapy, and viral suppression) has been utilized as a means for identifying care gaps and setting goals in patients with human immunodeficiency virus (HIV) infection, HCV-HIV co-infection, and recently for HCV mono-infected individuals as well [11–15]. Recently, barriers to completion of therapy have been reported which include but are not limited to diagnosis, knowledge of treatment options, completion of pre-treatment paperwork, lack of insurance coverage, medical eligibility, lack of program infrastructure for vulnerable populations, and medication costs [16–20]. To this effect, some interventions to improve access have also been proposed, such as provision for self-referral and shortened treatment duration [21, 22].

Among the steps in HCV treatment provision, pre-authorization (also known as prior authorization, prior approval, or pre-certification) is the process by which a health insurance provider determines that specific treatment is medically necessary, and which allows for insurance coverage of treatment cost. It is currently known that DAA therapy is expensive with prices ranging from $63,000 to $300,000 per treatment course. The wholesale cost of a 12-week treatment course of SOF/LED is $94,500, amounting to $1,125 per pill [23]. As this results in prohibitive cost and limited availability, pre-authorization often requires that patients have advanced fibrosis (grade F3 or beyond) or cirrhosis to be given treatment priority [24].

Currently there are limited data on rate and timing of insurance pre-authorization after SOF/LED prescriptions are written. In this study, we aim to perform a retrospective observational study reporting real-life data of drug approval rates in a cohort of patients with HCV infection who received prescription for SOF/LED treatment over a 3-month period. We also aim to determine factors associated with pre-authorization approval, time to pre-authorization decision, and time to pre-authorization approval. We hypothesize that the majority of patients for whom a pre-authorization request is filed will ultimately receive approval, and that insurance pre-authorization will be within the recommended guidelines for treatment for those with the highest need (i.e. advanced liver disease). However, we also hypothesize that there will be a proportion of patients who are ultimately not approved, as well as some who are approved only after appeal.

## Materials and Methods

### Study Subjects

As part of the SOF/LED acquisition process, all patients had pre-authorization requests sent to their insurance coverage providers. We reviewed the medical charts of all patients at Yale Liver Center who had an insurance pre-authorization request for SOF/LED filed between October 11, 2014 and December 31, 2014. Patients were then excluded if they received a prescription for HCV treatment other than combination SOF/LED.

### Outcomes

For each patient, we recorded the insurance provider of pre-authorization request. Those without Medicare or Medicaid insurance carriers were categorized as having private insurance coverage. If a patient was listed as having both Medicaid/Medicare and another insurance provider, they were considered to have a private insurance provider. We recorded approval, denial, or pending status of pre-authorization initial request and appeal as of March 1^st,^ 2015. If an individual was denied treatment and appeal was sought, date of appeal request and date of appeal decision were recorded.

### Covariates

Patient characteristics included age, race, body mass index, co-morbid hypertension, psychiatric illness, diabetes, renal disease, hepatitis B or HIV co-infection, and baseline biochemical markers (total bilirubin, serum creatinine, and serum international normalized ratio, INR). We recorded HCV viral characteristics, including genotype, viral load, IL28B gene variant and prior treatment regimens. Severity of HCV infection was determined by progression of hepatic fibrosis. Those with METAVIR stage 4 fibrosis on liver biopsy, clinical hepatic decompensation, or imaging findings suggesting cirrhosis with portal hypertension were classified as having cirrhosis. Advanced fibrosis included those with cirrhosis and included individuals with grade 3 fibrosis on liver biopsy, advanced fibrosis by tissue elastography, and/or an elevated FIB-4 score (>3.25) [25]. In patients with cirrhosis, Child-Pugh class and MELD scores were recorded using laboratory testing closest to the pre-authorization filing date. A subset of patients received their pre-authorization request through their transplant clinic provider. It was noted for those whom this was the case.

### Statistical Analysis

T-testing was used to compare continuous variables and chi-square testing was used for categorical variables. Univariate and multivariate analyses were performed using linear and logistic regression modeling with forward selection logistic regression to identify significant predictors of pre-authorization approval and times-to-decision or approval. All data were analyzed using SAS 9.4 statistical software (Cary, NC). Full dataset with SAS code used for this analysis is available at S1 Appendix.

### Ethics Statement

We obtained approval for conduct of this study by our institutional review board.

## Results

A total of 174 patients with chronic HCV infection seen at the Yale Liver Center were prescribed antiviral therapy between October 11^th^ and December 31^st^ 2014, of whom 129 were prescribed SOF/LED. Tables 1–3 summarize demographic characteristics of this patient population. The mean age was 57.0 ± 9.9 years with 61.2% being males. 60.5% of the population had cirrhosis. Table 4 summarizes the outcomes of pre-authorization. Of the 128 for whom pre-authorization status was determined, 100 (77.5%) received initial approval for pre-authorization. 117 (91.4%) of 129 received approval including those who required appeal. Initially, 19 patients (14.7%) required appeal and ultimately 6 (4.7%) were denied. As of March 1^st^, 2015, the pre-authorization status of 5 (3.9%) are pending and 1 (0.0%) is unknown. The average time to final decision (approval or denial) was 26.1 ± 25.2 days, and in those approved the average time to decision was 22.9 ± 21.2 days.

**Table 1:** Baseline demographic information for patients prescribed SOF/LED from Yale non-transplant hepatology and transplant hepatology clinics from October 1, 2014 to December 30, 2014 (n = 129).

Characteristic		Value
	Age in years, mean ± SD	57.0 ± 9.9
Gender, n (%)	Male	79 (61.2)
	Female	50 (38.8)
Race, n (%)	White	88 (68.2)
	Black	25 (19.4)
	Asian	4 (3.1)
	Other	11 (8.5)
	Unknown	1 (0.8)
Ethnicity, n (%)	Non-Hispanic	106 (82.2)
	Hispanic	21 (16.3%)
	Patient refused/Unk	2 (1.6%)
Medical insurance, n (%)	Private	63 (49.2)
	Public	64 (50.0)
	Unknown / None	1 (0.8)
Smoking History, n (%)	Non-smoker	29 (22.5)
	Prior smoker	54 (41.9)
	Active smoker	39 (30.2)
	Unknown	7 (5.4%)
Alcohol use history, n (%)	Never	36 (27.9)
	Occasional	29 (22.5)
	Prior abuse	42 (32.6%)
	Unknown	22 (17.1%)
Illicit drug use history, n (%)	Never	37 (28.7)
	Prior use	68 (52.7)
	Active use	6 (4.7)
	Unknown	18 (14.0)
	Body mass index, mean ± SD	29.0 ± 6.4
	Diabetes mellitus, n (%)	31 (24.0)
	Hypertension, n (%)	58 (45.0)
	Psychiatric history, n (%)	50 (38.8)
	HBV, n (%)	0 (0)
	HIV, n (%)	3 (2.3)
GFR, no (%)	>60	115 (89.2)
	≤60	14 (10.9)
	Followed in transplant clinic, n (%)	34 (26.4)

**Table 2:** Hepatitis C virus characteristics and disease severity.

Characteristic		Value
	Mean viral load, mean ± SD	2,960,146 ± 4,226,850
	Log10 (mean viral load)	6.47
Genotype, n (%)	1A	96 (74.4)
	1B	17 (13.2)
	1 subtype unspecified	12 (9.3)
	Non-GT1	2 (1.6)
	Unknown	2 (1.6)
IL28B polymorphism, n (%)	CC	21 (16.3)
	CT	44 (24.1)
	TT	19 (14.7)
	Unknown	45 (34.9)
	Prior HCV treatment, n (%)	57 (44.2)
	Multiple prior HCV treatments, n (%)	22 (17.1)
	Presence of advanced fibrosis, n (%)	89 (69.0)
	Presence of cirrhosis, n (%)	78 (60.5)

**Table 3:** Baseline characteristics of patients with cirrhosis (n = 78).

Characteristic		Value
	MELD score, mean ± SD	8.2 ± 2.6
Child-Pugh class, n (%)	A	58 (74.4)
	B	20 (25.6)
	Presence of decompensated cirrhosis, n (%)	26 (20.2)
	Presence of ascites, n (%)	17 (21.8)
	Presence of encephalopathy, n (%)	18 (23.1)
	Presence of prior variceal bleed, n (%)	11 (14.1)
	Presence of jaundice, n (%)	3 (3.9)
	Presence of hepatocellular carcinoma, n (%)	18 (23.1)
Transplant status, n (%)	Not indicated	46 (59.0)
	Not eligible	6 (7.7)
	Under evaluation	10 (12.8)
	Listed	9 (11.5)
	Post-transplant	7 (9.0)

**Table 4:** Pre-authorization outcomes for patients prescribed SOF/LED between October 11^th^-Dec 31^st^, 2014 (as of March 1^st^ 2015).

Characteristic		Value
	Total for whom outcomes data available, n	129
Final pre-authorization decision, n (%)	Approval	117 (91.4)
	Denial	6 (4.7)
	Pending	5 (3.9)
	Unknown	1 (0)
Initial pre-authorization decision, n (%)	Approval	100 (77.5)
	Denial or pending	24 (18.6)
	Time to decision in days, mean ± SD, (n)	26.1 ± 25.2 (126)
	Time to approval in days, mean ± SD, (n)	22.9 ± 21.2 (117)
	Time to denial in days, mean ± SD, (n)	32.8 ± 20.2 (4)
	Appeal required, n (%)	19 (14.7)
Result of appeal, n (%)	Approval	17 (89.5)
	Denial	1 (5.3)
	Approval	1 (5.3)
	Time of appeal process in days, mean ± SD, (n)	18.6 ± 22.1 (18)

Table 5 summarizes the time-to-decision in all subjects with outcomes data and time-to-approval in those who were approved for pre-authorization. Females were found to have a significantly lower time-to-decision than males (19.8 vs. 30.0 days, p = 0.01) with a similar but non-significant finding in time-to-approval. Those with a Medicare/Medicaid had a shorter average time-to-decision and time-to-approval though this finding was not significant (22.6 vs. 28.7 days, p = 0.18 & 19.2 vs. 25.9 days, p = 0.08, respectively). Those with Child-Pugh class B cirrhosis had a significantly shorter approval time (14.4 vs. 24.7 days, p = 0.048). Similar, non-significant findings were noted with those with advanced fibrosis and decompensated cirrhosis. Pre-authorization requests from liver transplant clinic were found to have a faster average time-to-decision and time-to-approval than pre-authorization requests from other clinics (17.9 vs. 28.9 days, p = 0.03 & 14.8 vs. 25.6 days, p = 0.02, respectively).

**Table 5:** Time-to-decision and time-to-approval in patients receiving SOF/LED therapy.

Characteristic		Time to Decision			Time to Approval		
		n	Time in days, mean ± SD	p-value	n	Time in days, mean ± SD	p-value
Age in years	≥60	71	29.7 ± 29.2	0.055	53	20.2 ± 16.6	0.187
	<60	55	21.4 ± 18.2	0.055	64	25.2 ± 24.3	0.187
Gender	Male	77	30.0 ± 28.7	0.01	69	25.3 ± 23.6	0.128
	Female	49	19.8 ± 16.9	0.01	48	19.6 ± 16.9	0.128
Race	White	86	26.6 ± 26.8	0.72	79	22.2 ± 20.8	0.57
	Other	40	24.9 ± 21.8	0.72	38	24.6 ± 22.3	0.57
	Black	24	26.8 ± 22.4	0.88	23	26.7 ± 22.9	0.35
	Other	102	25.9 ± 25.9	0.88	94	22.0 ± 20.8	0.35
	Hispanic	21	26.0 ± 22.6	0.99	20	25.7 ± 23.1	0.53
	Other	105	26.1 ± 25.8	0.99	97	22.4 ± 20.9	0.53
Insurance	Private	63	28.7 ± 24.0	0.18	55	25.9 ± 20.3	0.08
	Public	63	22.6 ± 25.2	0.18	60	19.2 ± 20.2	0.08
Cirrhosis	Yes	77	25.6 ± 23.3	0.81	72	22.9 ± 20.9	0.98
	No	49	26.7 ± 28.2	0.81	45	23.0 ± 21.9	0.98
Advanced Fibrosis	Yes	87	23.2 ± 22.6	0.08	82	20.6 ± 20.1	0.07
	No	39	32.5 ± 29.6	0.08	35	28.4 ± 23.2	0.07
Prior HCV Treatment	Yes	56	27.4 ± 26.7	0.60	49	21.7 ± 21.3	0.60
	No	70	25.0 ± 24.1	0.60	68	23.8 ± 21.3	0.60
Multiple prior treatments	Yes	22	31.4 ± 28.6	0.28	19	27.3 ± 27.4	0.33
	No	104	25.0 ± 24.4	0.28	98	22.1 ± 19.9	0.33
Decompensated cirrhosis	Yes	26	19.5 ± 22.8	0.14	25	17.1 ± 19.6	0.12
	No	100	27.8 ± 25.6	0.14	92	24.5 ± 21.5	0.12
Viral load	≥6M	16	33.6 ± 35.7	0.36	14	27.6 ± 29.0	0.52
	<6M	110	25.0 ± 23.3	0.36	103	22.3 ± 20.1	0.52
Transplant clinic	Yes	32	17.9 ± 20.6	0.03	29	14.8 ± 17.7	0.02
	No	94	28.9 ± 26.1	0.03	88	25.6 ± 21.7	0.02
GFR	>60	112	26.5 ± 24.9	0.58	104	23.5 ± 20.9	0.39
	≤60	14	22.6 ± 28.5	0.58	13	18.2 ± 24.2	0.39
Child-Pugh class	A	105	27.8 ± 25.2	0.09	97	24.7 ± 21.1	0.048
	B	21	17.5 ± 24.2	0.09	20	14.4 ± 20.1	0.048
HIV co-infection	Yes	3	11.0 ± 5.3	0.30	3	11.0 ± 5.3	0.33
	No	123	26.4 ± 25.4	0.30	114	23.3 ± 21.4	0.33

Table 6 summarizes proportions of unapproved and initially approved for those whom pre-authorization was sent categorized by patient characteristics. A significantly higher proportion of patients with Medicare/Medicaid were initially approved compared to those with private insurance (92.2% vs. 71.4%, p = 0.002). In addition, a significantly higher proportion of patients with a viral load ≥6 million were initially approved compared to individuals with viral load < 6 million (84.1% vs. 62.5%, p = 0.04).

**Table 6:** Disapproval and initial-approval rates in patients receiving SOF/LED preauthorization request.

Characteristic		Unapproved, n (%)	Initially Approved, n (%)	Chi-square	P-value
Age	≥60 years	61 (58.1)	44 (41.9)	1.19	0.28
	<60 years	11 (45.8)	13 (54.2)	1.19	0.28
Gender	Male	17 (21.5)	62 (78.5)	1.14	0.29
	Female	7 (14)	43 (86)	1.14	0.29
Race	White	19 (21.6)	69 (78.4)	N/A	0.09
	Other	5 (12.2)	36 (87.8)	N/A	0.09
	Black	4 (16.0)	21 (84.0)	N/A	0.22
	Other	20 (19.2)	84 (80.8)	N/A	0.22
	Hispanic	4 (19.1)	17 (81.0)	N/A	0.24
	Other	20 (18.5)	88 (81.5)	N/A	0.24
Insurance	Private	18 (28.6)	45 (71.4)	N/A	0.002
	Public	5 (7.8)	59 (92.2)	N/A	0.002
Cirrhosis	Yes	13 (16.7)	65 (83.3)	0.49	0.48
	No	11 (21.6)	40 (78.4)	0.49	0.48
Advanced Fibrosis	Yes	14 (15.7)	75 (84.3)	1.57	0.21
	No	10 (25.0)	30 (75.0)	1.57	0.21
Prior HCV Treatment	Yes	14 (24.6)	43 (75.4)	2.39	0.122
	No	10 (13.9)	62 (86.1)	2.39	0.122
Multiple prior treatments	Yes	7 (31.8)	15 (68.2)	3.06	0.08
	No	17 (15.9)	90 (84.1)	3.06	0.08
Decompensated cirrhosis	Yes	2 (7.7)	24 (92.3)	N/A	0.07
	No	22 (21.4)	81 (78.6)	N/A	0.07
Viral load	≥6M	6 (37.5)	10 (62.5)	4.31	0.04
	<6M	18 (15.9)	95 (84.1)	4.31	0.04
Transplant clinic	Yes	6 (17.7)	28 (82.4)	0.03	0.867
	No	18 (19.0)	77 (81.1)	0.03	0.867
Renal function	GFR >60	22 (19.1)	93 (80.9)	N/A	0.276
	GFR ≤60	2 (14.3)	12 (85.7)	N/A	0.276
Child-Pugh class	A	21 (19.4)	87 (80.6)	N/A	0.221
	B	3 (14.3)	18 (85.7)	N/A	0.221
HIV co-infection	Yes	0 (0)	3 (100)	N/A	0.40
	No	23 (19.1)	102 (81.0)	N/A	0.40

Univariate linear regression modeling results are shown in Table 7. Significant associations to shorter times-to-decision and times-to-approval were noted with psychiatric disease, high FIB-4 score, and pre-authorization request from transplant clinic. Also, significantly shorter times were noted with increases in total bilirubin, INR, FIB-4 score, and MELD score. Table 8 summarizes univariate logistic regression model results. This analysis revealed that having Medicare/Medicaid (OR 4.72, 95% CI 1.63–13.67) and a high viral load (OR 3.17, 1.02–9.81) were associated with higher odds of initial approval compared to private insurance and low viremia, respectively.

**Table 7:** Univariate linear regression analysis with time-to-decision and time-to-approval.

	Variable	Time-to-Decision (n = 126)		Time-to-Approval (n = 117)	
		Parameter estimate	p-value	Parameter estimate	p-value
Continuous Variables	Age	-0.35	0.117	-0.19	0.348
	AST	-0.01	0.848	-0.06	0.186
	ALT	-0.08	0.143	0.02	0.698
	Alkaline phosphatase	-0.004	0.922	0.001	0.976
	Total bilirubin	-8.71	0.036	-7.55	0.032
	Creatinine	2.61	0.740	0.11	0.987
	Platelets	0.03	0.291	0.05	0.055
	INR	-38.62	0.022	-37.78	0.028
	FIB-4 score	-0.93	0.031	-0.89	0.014
	MELD	-2.39	0.013	-2.10	0.011
	Viral load in millions	0.41	0.445	0.46	0.311
	Log_10_(viral load)	4.81	0.076	4.76	0.040
Dichotomous Variables	Private insurance	6.02	0.176	6.65	0.081
	Hypertension	-0.64	0.888	5.11	0.196
	Psychiatric disease	-3.60	0.435	-8.84	0.028
	Antecedent HCV treatment	2.38	0.601	-2.09	0.602
	Multiple prior HCV treatments	6.41	0.281	5.21	0.330
	High FIB-4 (>3.25) score	-6.89	0.140	-10.03	0.013
	Any cirrhosis	-1.09	0.815	-0.08	0.984
	Decompensated cirrhosis	-8.28	0.137	-7.46	0.120
	Transplant clinic	-10.99	0.033	-10.80	0.017

**Table 8:** Univariate logistic regression analysis for initial approval.

Variable	Odds ratio (95% CI)	p-value
Age ≥ 60 (vs. <60yo)	1.64 (0.67–4.00)	0.278
Public insurance	4.72 (1.63–13.67)	0.004
Hypertension	1.94 (0.79–4.77)	0.148
Psychiatric disease	0.35 (0.12–1.01)	0.052
Antecedent HCV treatment	2.02 (0.82–4.96)	0.126
Multiple prior HCV treatments	2.47 (0.88–6.96)	0.087
High FIB-4 (>3.25) score	0.67 (0.26–1.76)	0.414
Advanced fibrosis (F3-4)	0.56 (0.22–1.40)	0.214
Any cirrhosis (F4)	0.72 (0.30–1.78)	0.485
Decompensated cirrhosis	0.30 (0.07–1.40)	0.127
Transplant clinic	0.92 (0.33–2.54)	0.867
Viral load ≥6 M (vs. <6 M)	3.17 (1.02–9.81)	0.046
White race	1.98 (0.68–5.75)	0.208
Black race	0.80 (0.25–2.59)	0.710
Hispanic ethnicity	1.04 (0.31–3.41)	0.954
GFR < 60 (vs. GFR ≥60)	1.41 (0.30–6.80)	0.662

Multivariate linear and logistic models are shown in Tables 9–16. In multivariate linear models, forward stepwise addition revealed that MELD score, female gender, and advanced fibrosis were significant predictors of a shorter time-to-decision and time-to-approval, while psychiatric disease was found to be a significant predictor of a shorter time-to-approval. These associations were persistent after controlling for age and race (Tables 12–14). Forward stepwise selection logistic regression modeling revealed that having Medicare/Medicaid (OR 5.96, 95% CI 1.66–21.48) and viral load ≥6 million IU/mL (OR 4.54, 95% CI 1.08–19.08) were significant predictors of initial approval and persisted after controlling for age, gender, race, presence of cirrhosis or hypertension, and pre-authorization request from transplant clinic (Tables 15 and 16).

**Table 9:** Multivariate analyses for time-to-decision (n = 123). Stepwise linear regression model for time-to-decision.

Variable	Parameter Estimate	Partial R^2^	F-statistic	P-value
MELD score	-2.41	0.057	7.32	0.0078
Male Gender	13.08	0.051	6.86	0.0099
Advanced Fibrosis	-9.61	0.029	4.06	0.0462

Total model R^2^ = 0.137, F-value = 6.32, p = 0.0005

**Table 10:** Multivariate analyses for time-to-decision (n = 123). Prediction of time-to-decision based on linear multivariate model with selected variables (from stepwise selection as above) and demographic covariates (n = 123).

Variable	Parameter Estimate (SE)	t-value	p-value
Advanced Fibrosis	-11.17 (4.9)	-2.27	0.025
Male gender	11.38 (4.67)	2.42	0.017
Age (≥60 years)	-6.51 (4.52)	-1.44	0.152
White race	4.08 (7.65)	0.53	0.595
Black race	4.95 (9.04)	0.55	0.585
Hispanic	0.99 (6.78)	0.15	0.884

Model R^2^ = 0.104, F-value = 2.29, p = 0.040

**Table 11:** Multivariate analyses for time-to-decision (n = 123). Prediction of time-to-decision based on linear multivariate model with selected variables (from stepwise selection as above) and demographic covariates (n = 123).

Variable	Parameter Estimate (SE)	t-value	p-value
MELD score	-2.72 (0.97)	-2.80	0.006
Male gender	11.25 (4.60)	2.45	0.016
Age (≥60 years)	-5.84 (4.49)	-1.30	0.196
White race	6.70 (8.56)	0.89	0.377
Black race	5.80 (8.94)	0.65	0.518
Hispanic	6.36 (6.73)	0.94	0.347

Model R^2^ = 0.154, F-value = 4.99, p = 0.0010

**Table 12:** Multivariate model for time-to-approval (n = 117). Models included in final model after stepwise linear regression modeling.

Variable	Parameter Estimate	Partial R^2^	F-statistic	P-value
MELD score	-2.17	0.068	8.30	0.0047
Prior psychiatric disease	-8.17	0.039	4.92	0.0286
Gender (1 = male)	7.68	0.023	2.97	0.0877
Advanced fibrosis	7.83	0.028	3.64	0.0591

Total model R^2^ = 0.159, F-value = 5.19, p = 0.0007

**Table 13:** Multivariate model for time-to-approval (n = 117). Prediction of time-to-approval based on linear multivariate model with selected variables (from stepwise selection as above) and demographic covariates (n = 117).

Variable	Parameter Estimate (SE)	t-value	p-value
MELD score	-2.38 (0.84)	-2.83	0.0014
Prior psychiatric disease	-8.67 (3.93)	-2.20	0.0298
Gender (1 = male)	7.10 (3.92)	1.81	0.0723

Model R^2^ = 0.147, F-value = 2.67, p = 0.0136

Model in this table was controlled for: age, gender, race

**Table 14:** Multivariate model for time-to-approval (n = 117). Prediction of time-to-approval based on linear multivariate model with selected variables (from stepwise selection as above) and demographic covariates (n = 117).

Variable	Parameter Estimate (SE)	t-value	p-value
Advanced Fibrosis	-8.91 (4.42)	-2.02	0.0461
Prior psychiatric disease	-9.28 (4.00)	-2.32	0.0222
Gender (1 = male)	6.92 (4.02)	1.72	0.0879

Model R^2^ = 0.117, F-value = 2.06, p = 0.054

Model in this table was controlled for: age, gender, race

**Table 15:** Multivariate Logistic Modeling for proportion initially approved, (n = 123). Models included in final model after stepwise logistic regression modeling.

Variable	Chi-Square	p-value
Insurance provider	9.23	0.0024
Viral load (≥6M)	4.95	0.0262
Hypertension	3.19	0.0739

Included covariates: age, gender, race (3x binary variables: white vs. other, black vs. other, Hispanic vs. non-hispanic), insurance (private vs. public), transplant clinic, viral load (≥6M vs. <6M), body mass index, multiple antecedent HCV treatments, meld score, hypertension, diabetes, psychiatric conditions, cirrhosis, advanced fibrosis, FIB-4 score, total bilirubin, INR, creatinine

**Table 16:** Multivariate Logistic Modeling for proportion initially approved, (n = 123). Logistic model with selected variables, including other clinically-relevant covariates, in predicting initial approval (n = 123).

Variable	Odds ratio (95% CI)	Chi-Square	p-value
Medicare or Medicaid Insurance provider	5.96 (1.66–21.48)	7.46	0.0063
Viral load (≥6M)	4.54 (1.08–19.08)	4.27	0.0388

Model controlled for age, gender, race, hypertension, presence of cirrhosis, transplant clinic (measures of covariate associations not listed).

## Discussion

In our cohort of patients receiving pre-authorization request for SOF/LED over a three-month period, we found that nearly one in four were denied initial approval, although most patients eventually obtained drug authorization through the appeals process. Female gender, advanced Child-Pugh class, and liver transplant clinic were associated with shorter decision or approval times. Finally, having Medicare/Medicaid and a high viral load were significant predictors for initial approval, with findings persisting after controlling for demographic covariates.

The cascade of care model for HCV treatment involves numerous steps from diagnosis to successful treatment and viral eradication with patient drop-out observed at every step [11]. This analysis focused on one specific process: pre-authorization request and approval in those with a known diagnosis of HCV infection prescribed SOF/LED. Fewer than 10% patients ultimately failed to obtain access to therapy, although the appeals process led to further delay to treatment initiation. Importantly, the proportion of patients with access to drug therapy may be overestimated as this analysis was largely restricted to insured patients, all of whom had already successfully linked to specialty care in a major tertiary care university liver clinic, completed a series of pre-treatment evaluations and a formal structured HCV class, and were deemed by a specialty provider to represent an appropriate candidate with adequate motivation to initiate treatment.

The higher approval rates in patients with Medicare/Medicaid was unexpected, and could not be explained by other patient or medical variables, as this association remained significant in the multivariate model. Following Food and Drug Administration (FDA) approval of SOF/LED on October 10, 2014, updates to AASLD-IDSA HCV treatment recommendations affirmed that treatment be considered for all patients regardless of disease severity, although with the highest priority given to patients with advanced fibrosis, transplant recipients, or those with severe renal insufficiency [3]. Our hypothesis is that the higher than expected authorization rates by Medicare/Medicaid represented a time-limited anomaly driven by the absence of prior authorization guidelines until December 2014 and January 2015, through which Harvoni has been restricted by state Medicaid to patients with advanced liver fibrosis or cirrhosis (F3/F4), and selected patients at high risk for disease progression, and must be prescribed by specialty physicians [26, 27]. Restrictive prior authorization guidelines were established by many public and private payors in this state by early 2015 (Table 17). As nearly half of patients prescribed SOF/LED in this analysis had Medicare/Medicaid coverage, drug authorization rates would be expected to be lower beyond January 2015.

**Table 17:** Select information requested for pre-authorization for specified insurance providers. Exact criteria should be found in appropriate insurance pre-authorization form.

	HCV Genotype/Subtype	Viral Load	Presence of advanced fibrosis or cirrhosis	Presence of hepatic decompensation	Mechanism of fibrosis staging and result	Liver transplant recipient	Non liver transplant recipient	Presence of ESRD	Cryoglobulinemia or glomerular disease	HIV co-infection +/- viral load count	HBV co-infection	Prior sofosbuvir treatment and response	Other prior HCV treatment & response	Drug/alcohol use	Prescriber specific criteria
Accredo	x	x											x		
Aetna/Open Choice	x	x	x	x	x	x	x	x		x	x		x		
Anthem	x	x	x	x	x	x		x	x			x	x	x	
AARP	x	x	x	x	x								x		x
Catamaran	x		x					x				x			x
Cigna	x	x	x		x								x	x	x
Connecticare	x	x	x										x		
CVS Caremark	x	x										x	x		x
Medicare	x	x	x	x		x									x
Medicaid	x	x	x		x			x		x		x			x
Oxford	x			x	x	x		x		x			x	x	x
Tricare	x	x	x								x	x	x		x
United Health Care	x			x	x	x		x		x			x	x	x

In our cohort, patients in the liver transplant clinic were found to have shorter approval times, which may be attributable in part to overrepresentation of advanced liver disease in this population, and therefore likely be given initial approval through the pre authorization process with both public and private payors. We could not exclude the potential effect of variable access to certified specialty pharmacies with capacity to directly dispense SOF/LED medications to patients.

This is the first study to our knowledge assessing real-world access to interferon-free DAA regimens in established cohorts of patients with chronic HCV seeking antiviral therapy. These results contribute to the limited data available addressing proportion of patients successfully obtaining drug authorization through public and private insurance carriers, time to approval, and predictors for approval. Several limitations of our study warrant further investigation. We did not record data on proportion of treatment-eligible patients seeking treatment who declined to pursue SOF/LED prescription due to absence of insurance coverage, or perception of difficulty in accessing treatment due to mild liver fibrosis or other factors. Although the analysis was performed for consecutive unselected patients prescribed SOF/LED, this cohort represented a subset of patients who were deemed to be excellent candidates for treatment, and therefore selection bias by prescribing providers for individuals with anticipated approval could not be excluded. This study is also limited to authorization data in Connecticut, and state Medicaid and Medicare approval rates likely differ by states as well. Furthermore this study is focused exclusively on SOF/LED, and authorization results may be different for other FDA-approved interferon-free regimens such as sofosbuvir/simeprevir and paritaprevir/ritonavir, ombitasvir, dasabuvir, and ribavirin. Future studies are needed to clarify the variance in public and private insurance access to HCV regimens across states, stratified by liver fibrosis and other patient characteristics, the outcome of appeal requests, and approval of requests which are beyond FDA label or AASLD/IDSA recommendations.

In conclusion, we found that most patients filing a pre-authorization request for SOF/LED are eventually approved, but nearly 1 in 4 were denied access upon initial request, which may represent a barrier within the HCV care cascade. On multivariate analysis, advanced liver disease was associated with faster approval time, while Medicare/Medicaid and high viremia were associated with insurance approval. Further studies are warranted to investigate the impact of evolving drug authorization policies by Medicare/Medicaid and private payers on access to curative HCV therapies such as SOF/LED.

## Supporting Information

S1 Appendix Full dataset with SAS code. (SAS)
